# Prediction of cognitive decline in Parkinson's disease based on MRI radiomics and clinical features: A multicenter study

**DOI:** 10.1111/cns.14789

**Published:** 2024-06-24

**Authors:** Yongjie Jian, Jiaxuan Peng, Wei Wang, Tao Hu, Jing Wang, Hui Shi, Xiaoyong Li, Jingfang Chen, Yuyun Xu, Yuan Shao, Qiaowei Song, Zhenyu Shu

**Affiliations:** ^1^ Jinzhou Medical University Postgraduate Training Base (Zhejiang Provincial People's Hospital, People's Hospital of Hangzhou Medical College) Hangzhou Zhejiang China; ^2^ Department of Radiology, Affiliated Hospital of Sichuan Nursing Vocational College The Third People's Hospital of Sichuan Province Chengdu Sichuan China; ^3^ Department of Radiology The First Affiliated Hospital of Chongqing Medical and Pharmaceutical College Chongqing China; ^4^ Department of Neurology, Affiliated Hospital of Sichuan Nursing Vocational College The Third People's Hospital of Sichuan Province Chengdu Sichuan China; ^5^ Department of Medical Technology Sichuan Nursing Vocational College Chengdu Sichuan China; ^6^ Center for Rehabilitation Medicine, Department of Radiology Zhejiang Provincial People's Hospital (Affiliated People's Hospital), Hangzhou Medical College Hangzhou Zhejiang China

**Keywords:** cognitive impairment, magnetic resonance imaging, models, Parkinson's disease, radiomics

## Abstract

**Objective:**

To develop and validate a multimodal combinatorial model based on whole‐brain magnetic resonance imaging (MRI) radiomic features for predicting cognitive decline in patients with Parkinson's disease (PD).

**Methods:**

This study included a total of 222 PD patients with normal baseline cognition, of whom 68 had cognitive impairment during a 4‐year follow‐up period. All patients underwent MRI scans, and radiomic features were extracted from the whole‐brain MRI images of the training set, and dimensionality reduction was performed to construct a radiomics model. Subsequently, Screening predictive factors for cognitive decline from clinical features and then combining those with a radiomics model to construct a multimodal combinatorial model for predicting cognitive decline in PD patients. Evaluate the performance of the comprehensive model using the receiver‐operating characteristic curve, confusion matrix, F1 score, and survival curve. In addition, the quantitative characteristics of diffusion tensor imaging (DTI) from corpus callosum were selected from 52 PD patients to further validate the clinical efficacy of the model.

**Results:**

The multimodal combinatorial model has good classification performance, with areas under the curve of 0.842, 0.829, and 0.860 in the training, test, and validation sets, respectively. Significant differences were observed in the number of cognitive decline PD patients and corpus callosum‐related DTI parameters between the low‐risk and high‐risk groups distinguished by the model (*p* < 0.05). The survival curve analysis showed a statistically significant difference in the progression time of mild cognitive impairment between the low‐risk and the high‐risk groups.

**Conclusions:**

The building of a multimodal combinatorial model based on radiomic features from MRI can predict cognitive decline in PD patients, thus providing adaptive strategies for clinical practice.

## Graphical abstract

The multimodal model based on MRI radiomics combined with clinical features, may serve as good predictors of cognitive decline in Parkinson's disease.

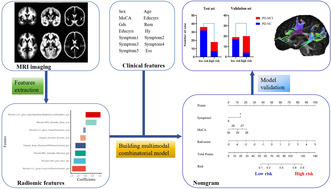

## 1 INTRODUCTION

Parkinson's disease (PD) is a common neurodegenerative disease that has become one of the fastest‐growing neurological diseases in terms of incidence rate, disability rate, and mortality, second only to Alzheimer's disease (AD),^1^ there are over 7 million people worldwide suffering from PD.^2^ Cognitive impairment is one of the more common nonmotor symptoms in PD, including mild cognitive impairment in PD (PD‐MCI) and PD dementia (PDD), where PD‐MCI is an independent risk factor for PDD.^3^ Epidemiological studies show that the incidence rate of PD‐MCI is high, up to 42.5%, and can appear in the early stage of PD.^4^ Among patients with more than ten years of medical history, the incidence rate of PDD accounts for 46%.^5^ When PD‐MCI progresses to PDD, it seriously affects patients' social function and quality of life. Therefore, the early diagnosis and intervention of PD‐MCI have crucial clinical significance.^6^

With the continuous development of medical imaging technology as indicated by MRI in neuroimaging, abnormal brain function, microanatomical structure, and pathological changes in PD patients have gradually been revealed. Changes in brain tissue microstructure precede morphological changes, but those microstructural changes cannot be observed by the naked eye on structural MRI (sMRI) images in early PD patients.^7^ Although functional MRI, such as neuromelanin‐sensitive MRI and quantitative susceptibility mapping (QSM), plays a vital role in the diagnosis of PD,^8^, ^9^ the diagnostic utility of these technologies highly depends on changes in scanners and acquisition protocols.^10^ Other methods, such as fluorodeoxyglucose positron emission tomography (FDG PET) and dopamine transporter single‐photon emission computed tomography (DAT‐SPECT), also play essential roles in diagnosing PD.^11^, ^12^ However, these are expensive models with relatively low accessibility, and they cannot be widely used in clinical practice. Therefore, developing a simple, noninvasive method to identify asymptomatic PD patients with potential cognitive decline is a massive challenge for clinical and imaging physicians.^13^

In recent years, the emergence of radiomics has provided a novel method for studying various neurodegenerative diseases, including PD.^14^ Radiomics can be used to transform digital medical images into high‐dimensional data that can be mined to support clinical decision‐making.^15^ Early radiomic features are often used in the study of tumor lesions. In recent years, an increasing number of studies have applied this method to diagnose neurodegenerative diseases, including auxiliary clinical diagnosis, treatment guidance, and progression prediction for PD.^16^ Research by Betrouni et al.^17^ has shown that before conventional MRI imaging methods detect brain tissue atrophy, radiomic features can already be used to detect differences in the brain between PD patients and healthy controls, suggesting that radiomics analysis can reflect the variation and distribution of local tissue characteristics in early stages of PD patients, capturing subtle structural changes. Our previous research was based on the use of T1WI structural images to identify radiomic features of the entire white matter of the brain, which have essential value for diagnosing PD.^18^ Based on the above research results, we assume that a radiomics model constructed using conventional T1WI can also be used to identify high‐risk populations in PD patients who may develop PD‐MCI, and their predictive performance can be improved by the addition of relevant clinical features.

This study aimed to extract features of the brain's entire gray and white matter from conventional T1WI images and construct a radiomics model for identifying PD‐MCI patients. Second, a multimodal combinatorial model was constructed based on radiomic features and relevant clinical features to predict high‐risk populations where PD normal control (PD‐NC) may progress to PD‐MCI.

## 2 MATERIALS AND METHODS

### 2.1 Patient information

The case data used in this study came from the PPMI and NACC databases. The Parkinson's Progression Markers Initiative (PPMI) (http://www.PPMI‐info.org) is the first global collaborative project composed of researchers, funders, and research participants dedicated to identifying biomarkers to improve PD treatment. It is a multicenter collaborative PD open‐source database with neuropsychological scales, MRIs, and genetic data. The National Alzheimer's Coordinating Center (NACC) (https://naccdata.org) was established in 1999; it is a large‐scale compilation of longitudinal data for healthy control (HC) participants and patients with mild cognitive impairment (MCI), AD, and other neurodegenerative diseases, including standardized clinical and neuropathological research data collected from AD centers across the United States.^19^, ^20^ For ethical review information on the data, please refer to the website. This study included 222 patients with a baseline diagnosis of PD‐NC. The inclusion criteria were as follows: all patients with an initial diagnosis of PD‐NC were followed up for 4 years, underwent MRI examinations, and had complete clinical data. The exclusion criteria were as follows: (1) the original MRI DICOM file was incorrect and we were unable to extract radiomic features; and (2) biological indicator and scale evaluation data were missing. All patients underwent brain MRI and detailed neuropsychological tests at the initial assessment. We randomly divided 183 patients from the PPMI database into a training set (*n* = 129) and a test set (*n* = 54) in a 7:3 ratio. Among them, 50 patients progressed to PD‐MCI during a 4‐year follow‐up period and were classified as the progression group, while the remaining 133 patients were classified as the nonprogression group. The 39 patients collected from the NACC database were used as an external validation set, of which 18 patients progressed to PD‐MCI during a 4‐year follow‐up period and were classified as the progression group, while the remaining 21 patients were classified as the nonprogression group. The specific screening process can be found in Figure S1.

**FIGURE 1:**
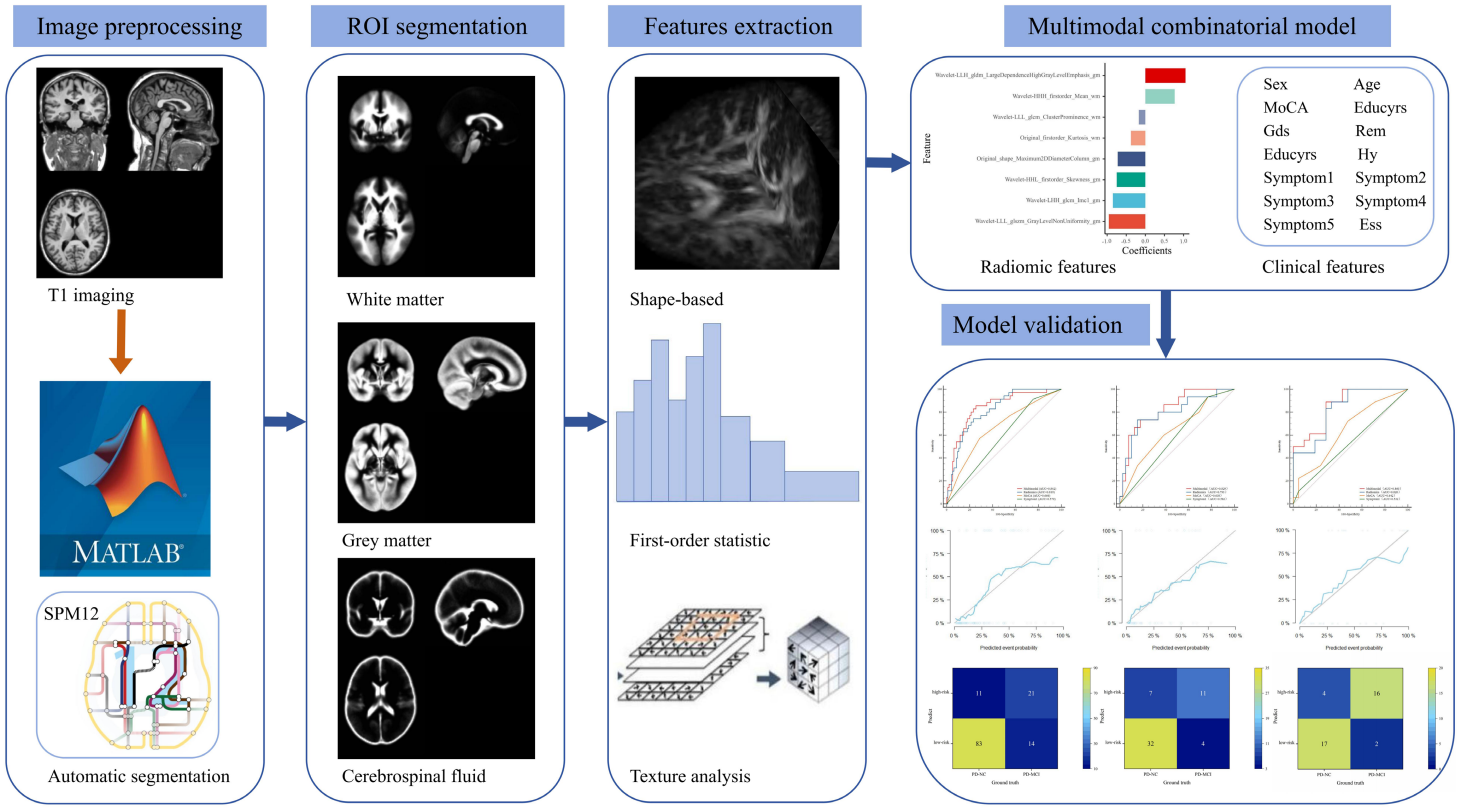
Architecture of the proposed PD progression prediction model.

We also collected the corresponding clinical data for this study, including neural scale information such as the Montreal Cognitive Assessment (MoCA) score, Epworth Sleepiness Scale score, and Geriatric Depression Scale score and clinical data such as age, sex, and education level. A multimodal combinatorial model was established using the training set, and the model's reliability was verified using the test set. In addition, to further validate the model's generalization performance, we used a validation set for model validation. The specific process is shown in Figure 1.

### 2.2 Radiomic features preprocessing

All experimental data were obtained through scanning with a 1.5T/3.0T MRI system, including T1WI images of all patients and DTI images of some patients. To further reduce the impact of image scanning parameters on feature extraction, we preprocessed the structural T1 image, including converting voxel values to 1 × 1 × 1 mm^3^. We standardized the image grayscale level to 1–32 levels to eliminate the influence of anisotropy on feature extraction.^21^ Afterward, the preprocessed images were imported into the statistical parameter mapping SPM12 software (version V2.5.5) on the MATLAB platform (MathWorks, MA, USA). The images were automatically segmented into gray matter, white matter, and cerebrospinal fluid (CFS) using the ITK‐SNAP software package (http://www.itksnap.org/pmwiki/pmwiki.php), with further manual correction; this includes (1) removing the nonbrain tissue, brainstem, and cerebellum and (2) correcting segmentation errors in brain tissue. The manual correction of MRI was independently performed by two experienced neuroradiologists who were unaware of the clinical data. After manual correction, the segmented brain tissue regions are imported as masks into PyRadiomics software for feature extraction.^22^ In addition, we selected DTI examinations from 52 PD patients for validation analysis in subsequent studies. The details of DTI feature extraction can be found in Data S1.

### 2.3 Radiomic features extraction

The PyRadiomics platform was used to extract radiomic features of the volume of interest (VOI). The extracted features include the following two categories: (1) original image features: ① 14 types of shape‐based features; ② 18 types of first‐order statistics; and ③ texture features, including 22 types of gray‐level cooccurrence matrix (GLCM) features, 16 types of gray‐level run‐length matrix (GLRLM) features, 16 types of gray‐level size zone matrix (GLSZM) features, and 14 types of gray‐level dependency matrix (GLDM) features; and (2) features related to the filter class: ① wavelet (WT) features; and ② Laplacian transforms, etc. A total of 3396 radiomic features were extracted, including 1132 gray matter (GM) features, 1132 white matter (WM) features, and 1132 CFS features. Detailed feature information can be found in Table S1. These features were extracted based on the region of interest and manually modified by radiologists to obtain the most consistent features among different radiologists, thereby ensuring robustness. The correlation coefficients (CCs) of each feature between feature set A (from radiologist A) and feature set B (from radiologist B) were calculated using Spearman rank correlation. Features with CC > 0.8 were considered robust features.^23^

### 2.4 Feature dimensionality reduction and composition of the radiomics model

To reduce the high‐dimensional burden on model training, the above features obtained from the training set were used to eliminate redundant and irrelevant features using minimum redundance maximum relevance (mRMR), and then the least absolute shrinkage and selection operator (LASSO) was used for dimensionality reduction processing, with the optimal parameters, ɑ, to construct a penalty function to eliminate variables with zero coefficient values. We used tenfold cross‐validation to obtain the optimal penalty parameter for LASSO and retained features with nonzero regression coefficients. Redundant or nonreproducible features were combined or excluded to screen out the most valuable and relevant features. Finally, based on the remaining radiomic features filtered out from the training set, logistic regression was used to construct a radiomics model, and the score values for each patient were calculated using the marker formula. The score values reflect the possible probability of PD‐NC progressing to PD‐MCI, which is called the Rad‐score. The area under the curve (AUC) of the receiver‐operating characteristic (ROC) curve was used to evaluate the accuracy of the radiomics model in the training, test, and validation sets. For detailed information on dimensionality reduction, please refer to the Data S2.

### 2.5 Construction and verification of a multimodal combinatorial model

The independent predictors were selected from clinical features and radiomics model in the training set by using the reverse stepwise selection method based on the Akaike information criterion (AIC), and a multimodal combinatorial model was established on this basis. To verify the improvement in model performance after including a radiomics model, we used the ROC curve and AUC to evaluate the performance of the multimodal combinatorial model, radiomics model, and related independent predictive factors. We used the Hosmer–Lemeshow test to analyze the goodness‐of‐fit of the multimodal combinatorial model and used calibration curves to visually evaluate the consistency between the predicted MCI probability and the actual MCI probability. In addition, we used Delong testing to determine the differences between the multimodal combinatorial and other model. Finally, the confusion matrix and F1 score were used to describe the performance of the multimodal combinatorial model.

### 2.6 Clinical validation

The model was constructed on the PPMI data, according to the optimal cutoff values in the training set, the training and test sets patients were divided into low‐risk and high‐risk groups, and the performance of the model on the NACC data was evaluated. The number of individuals progressing to PD‐MCI in the low‐risk and high‐risk groups was compared, and the difference in the MCI progression rate was examined. Considering the lack of biological interpretability of radiomic features, this study also included DTI parameters to verify the performance of biomarkers constructed from radiomic features.

### 2.7 Statistical analyses

All statistical analyses were conducted using R statistical software (v. 3.5.1), MedCalc software (V.11.2; 2011 MedCalc software bvba, Mariakerke, Belgium), and SPSS (software version 22, IBM, Armonk, NY, USA). The “mRMRe” package in R statistical software was used to filter the correlation and nonredundancy of the radiomic features. The LASSO logic in the “Glmnet” software package was used to select predicted features.

We tested the compliance of the quantitative data with normal distribution via the Kolmogorov–Smirnov test and Shapiro–Wilk test. Continuous variables for normal distribution were presented as the mean ± standard deviation and compared using the t‐test. For non‐normal distribution data, variables were expressed as medians (interquartile ranges, IQRs) and analyzed using Mann–Whitney *U* test. Categorical variables were recorded as frequencies (%), and the chi‐squared test or Fisher's exact test was used to evaluate the association of categorical variables. The metrics in the ROC curve (such as AUC, sensitivity, and specificity) and F1 score were used to evaluate model performance. All statistical data were bidirectional, and a *p* value less than 0.05 was considered statistically significant.

## 3 RESULTS

### 3.1 Comparison of clinical factors

The baseline clinical characteristics of 222 PD patients in the training, test, and validation sets are summarized in Table 1. In the training, test, and validation sets, 35 patients (27.1%), 15 patients (27.8%), and 18 patients (46.2%) developed PD‐MCI within 4 years after PD diagnosis, respectively. The majority of patients who developed PD‐MCI within the prescribed time window were male. Compared with patients without PD‐MCI, patients with PD‐MCI showed a higher incidence of Symptom1. In the training set, there were significant differences in Age and MoCA between the progression and nonprogression groups, with *p* values of 0.039 and 0.003, respectively. In addition, the test set showed a statistically significant difference in GDS between the progression and nonprogression groups, with *p* values of 0.021.

**TABLE 1:** Comparative analysis of clinical data for training, test, and validation sets.

Characteristics	PPMI						NACC		
	Training set (*n* = 129)			Test set (*n* = 54)			Validation set (*n* = 39)		
	Nonprogression (*n* = 94)	Progression (*n* = 35)	*p*‐Value	Nonprogression (*n* = 39)	Progression (*n* = 15)	*p*‐Value	Nonprogression (*n* = 21)	Progression (*n* = 18)	*p*‐Value
Sex (*n*, %)									
Male	55 (58.5%)	24 (68.6%)	0.401	28 (71.8%)	11 (73.3%)	1.000	10 (47.6%)	12 (66.7%)	0.232
Female	39 (41.5%)	11 (31.4%)		11 (28.2%)	4 (26.7%)		11 (52.4%)	6 (33.3%)	
Age (year), mean (SD)	62.6 ± 7.1	65.5 ± 7.0	0.039*	64.2 ± 7.2	66.3 ± 10.0	0.372	68.5 ± 6.0	72.4 ± 8.3	0.083
MoCA, median (IQR)	28.0 (27.0, 29.0)	27.0 (26.0, 28.0)	0.003**	28.0 (27.0, 29.0)	27.0 (26.0, 28.0)	0.108	29.0 (27.0, 30.0)	27.0 (24.5, 29.0)	0.069
GDS, median (IQR)	1.0 (0.0,2.0)	2.0 (1.0,3.0)	0.065	1.0 (0.0,2.0)	2.0 (1.0,3.0)	0.021*	2.0 (1.0,3.0)	2.0 (1.0,4.0)	0.568
Educyrs, median (IQR)	16.0 (14.0, 18.0)	16.0 (14.0, 18.0)	0.946	16.0 (15.0, 18.0)	16.0 (14.0, 18.0)	0.393	18.0 (16.0, 18.0)	18.0 (15.5, 18.5)	0.568
Symptom1 (*n*, %)									
No	23 (24.5%)	3 (8.6%)	0.079	9 (23.1%)	1 (6.7%)	0.318	20 (95.2%)	16 (88.9%)	0.458
Yes	71 (75.5%)	32 (91.4%)		30 (76.9%)	14 (93.3%)		1 (4.8%)	2 (11.1%)	
Symptom2 (*n*, %)									
No	26 (27.7%)	9 (25.7%)	1.000	8 (20.5%)	3 (20%)	1.000	NA^a^	NA^a^	NA
Yes	68 (72.3%)	26 (74.3%)		31 (79.5%)	12 (80%)				
Symptom3 (*n*, %)									
No	9 (9.6%)	5 (14.3%)	0.655	10 (25.6%)	4 (26.7%)	1.000	12 (57.1%)	8 (44.4%)	0.429
Yes	85 (90.4%)	30 (85.7%)		29 (74.4%)	11 (73.3%)		9 (42.9%)	10 (55.6%)	
Symptom4 (*n*, %)									
No	83 (88.3%)	31 (88.6%)	1.000	37 (94.9%)	13 (86.7%)	0.652	19 (90.5%)	12 (66.7%)	0.066
Yes	11 (11.7%)	4 (11.4%)		2 (5.1%)	2 (13.3%)		2 (9.5%)	6 (33.3%)	
Symptom5 (*n*, %)									
No	75 (79.8%)	31 (88.6%)	0.368	31 (79.5%)	13 (86.7%)	0.828	NA^a^	NA^a^	NA
Yes	19 (20.2%)	4 (11.4%)		8 (20.5%)	2 (13.3%)				
Hy (*n*, %)									
No	45 (47.9%)	14 (40%)	0.549	22 (56.4%)	6 (40.0%)	0.437	NA^a^	NA^a^	NA
Yes	49 (52.1%)	21 (60.0%)		17 (43.6%)	9 (60%)				
ESS (*n*, %)									
No	77 (81.9%)	32 (91.4%)	0.292	30 (76.9%)	13 (86.7%)	0.675	NA^a^	NA^a^	NA
Yes	17 (18.1%)	3 (8.6%)		9 (23.1%)	2 (13.3%)				
RBD (*n*, %)									
No	62 (66.0%)	20 (57.1%)	0.472	30 (76.9%)	8 (53.3%)	0.171	12 (57.1%)	11 (61.1%)	0.802
Yes	32 (34.0%)	15 (42.9%)		9 (23.1%)	7 (46.7%)		9 (42.9%)	7 (38.9%)	

Abbreviations: Age (year), Age at Enrollment; Educyrs, Years of Education; Symptom1, Initial symptom (at diagnosis)‐Resting Tremor; ESS, Epworth Sleepiness Scale Score; GDS, Geriatric Depression Scale Score; Hy, Hoehn & Yahr (OFF State); MoCA, Montreal Cognitive Assessment score (adjusted for education); NA, not available; RBD, Rapid Eye Movement Sleep Behavior Disorder Score; Symptom2, Initial symptom (at diagnosis)‐Rigidity; Symptom3, Initial symptom (at diagnosis)‐Bradykinesia; Symptom4, Initial symptom (at diagnosis)‐Postural Instability; Symptom5, Initial symptom (at diagnosis)‐Other.

^a^ Not available for all NACC study participants.

* *p* < 0.05.

** *p* < 0.01.

### 3.2 Construction and verification of radiomic markers

**FIGURE 2:**
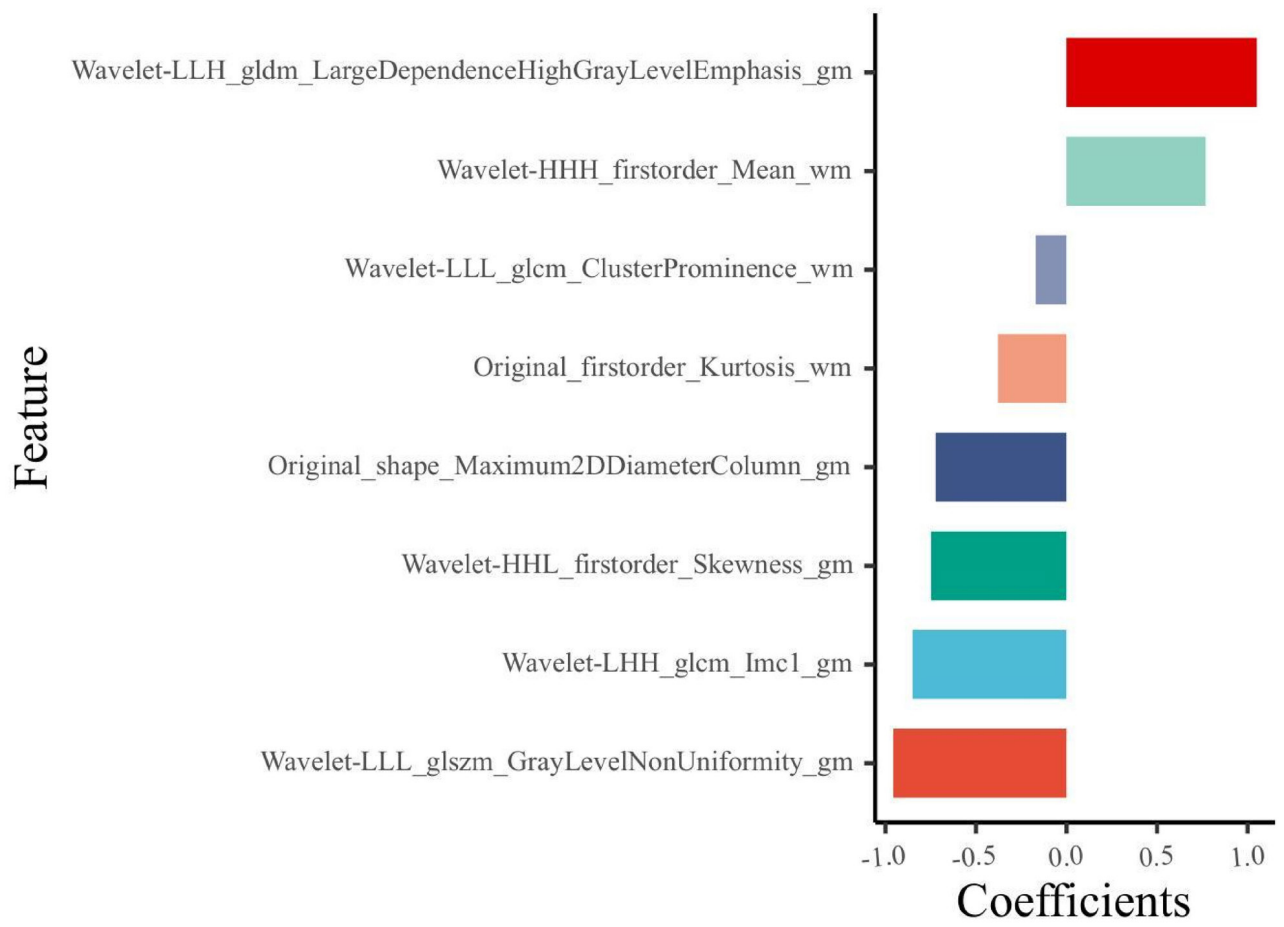
Feature weight histogram. The horizontal coordinates indicate the weight values of each feature.

**FIGURE 3:**
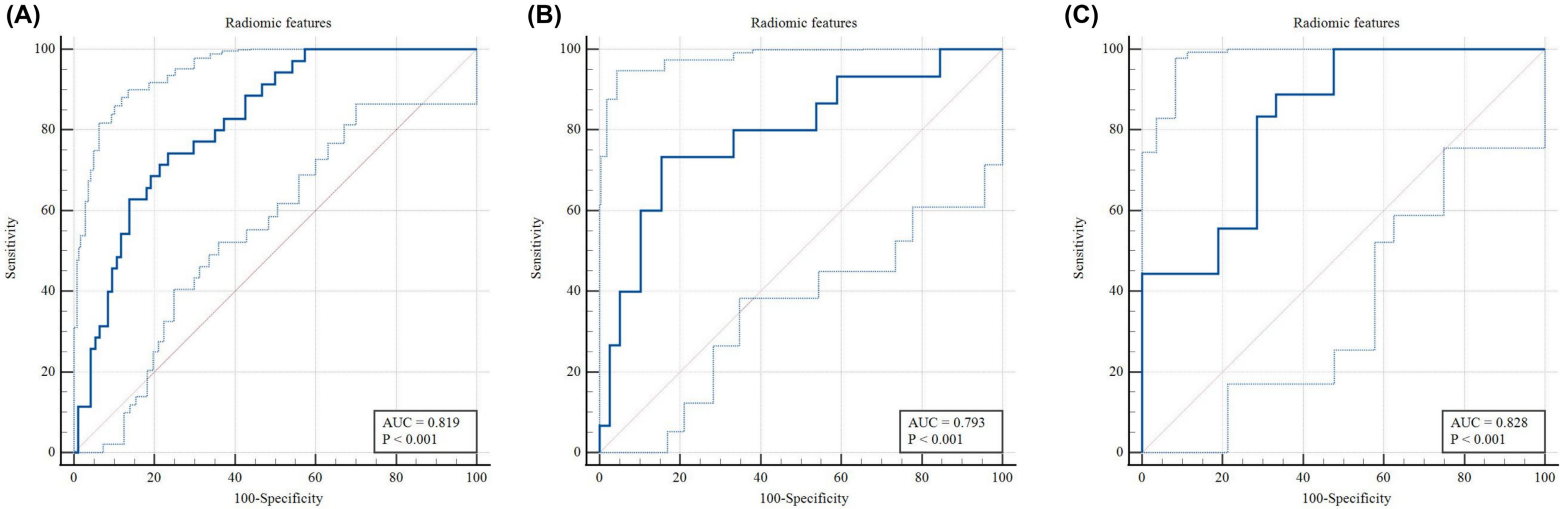
A, B, and C show the diagnostic performance of the radiomic features in the training, test, and validation sets, respectively.

After mRMR and LASSO dimensionality reduction, 8 radiomic features remained, including 5 GM and 3 WM features. The feature weights are shown in Figure 2. The radiomics model constructed based on these 8 features has good predictive performance on the training, test, and validation sets. The AUC values in the training, test, and validation sets were 0.819, 0.793, and 0.828, respectively, with sensitivity values of 0.743, 0.733, and 0.889 and specificity values of 0.766, 0.846, and 0.667, respectively (Figure 3).

### 3.3 Construction of a multimodal combinatorial model

**FIGURE 4:**
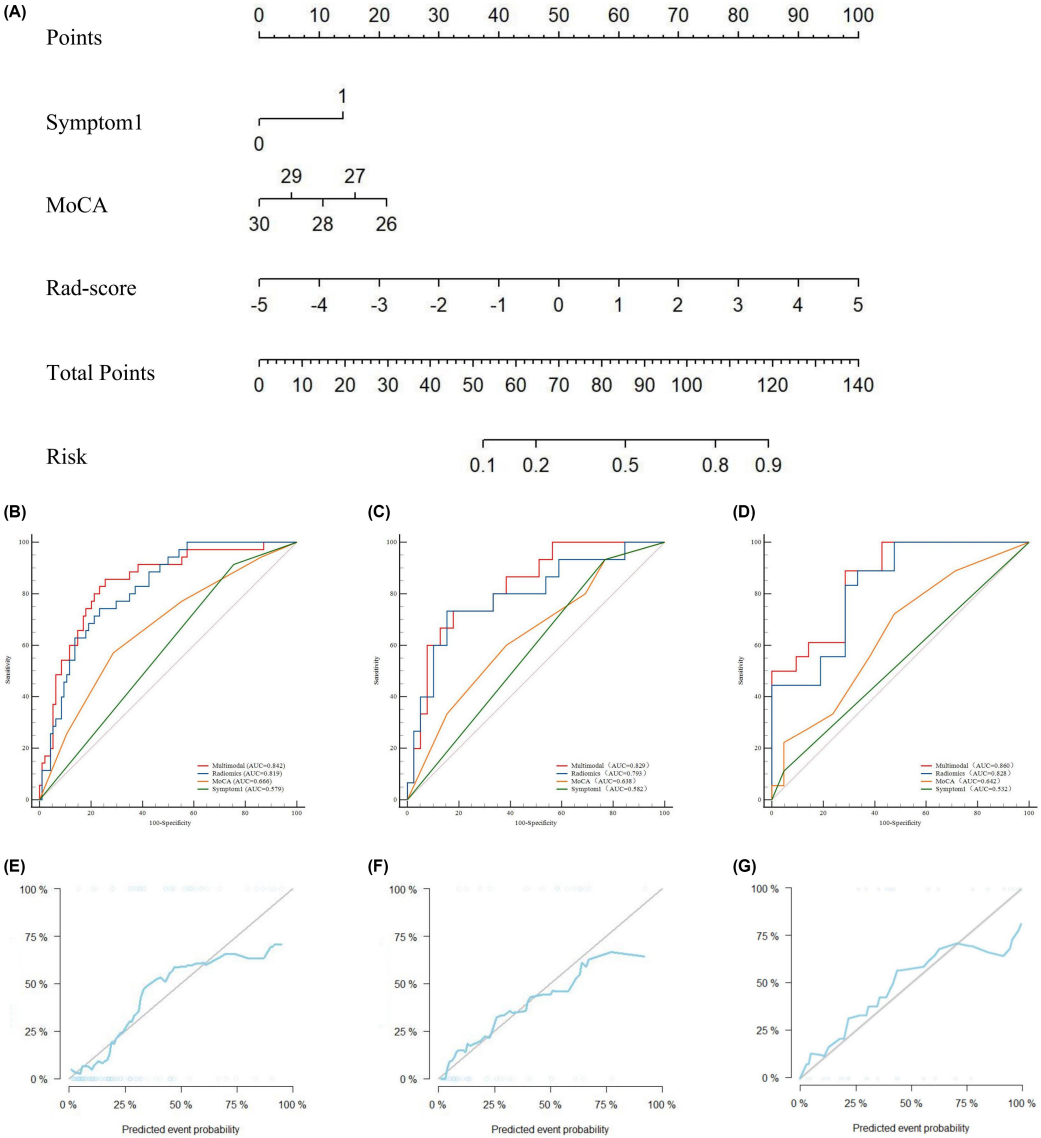
A shows the Radiomics nomogram for the prediction of PD‐MCI. The radiomics nomogram is developed in the training set with two clinical features (MoCA, Symptom1) and Rad‐score. B, C, and D show the diagnostic performance of the multimodal combinatorial model, radiomics model, MoCA score, and Symptom1 in the training, test, and validation sets, respectively. E, F, and G show the correction curves for the training, test, and validation sets, respectively.

**FIGURE 5:**
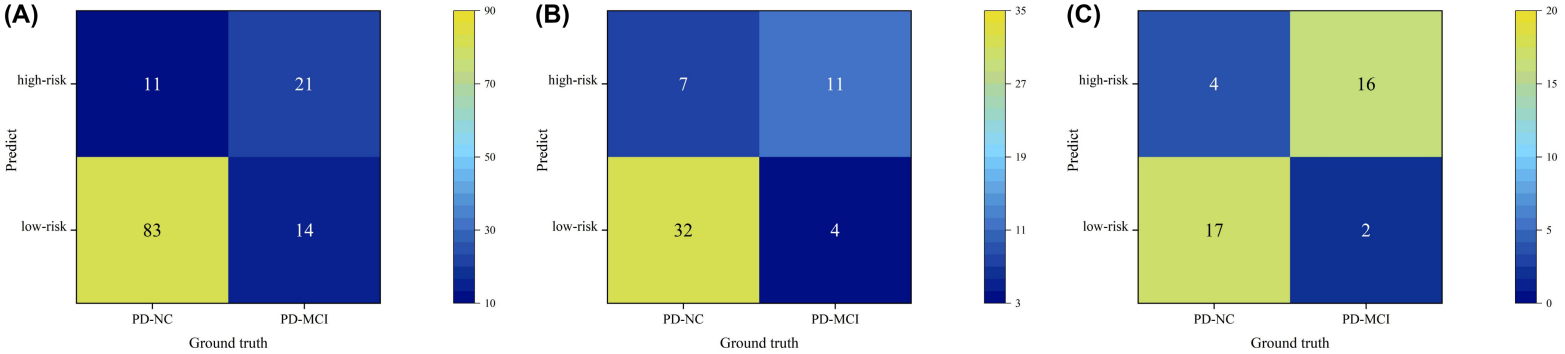
A, B, and C show the confusion matrix of the multimodal combinatorial model in the training, test, and validation sets, respectively.

Multivariate logistic regression showed that two clinical features (MoCA score, Symptom1) and Rad‐score were independent predictors of cognitive decline in PD patients. A multimodal combinatorial model was constructed based on the independent predictors, and a visual nomogram was established (Table 2 and Figure 4A). The nomogram scores were given based on the weights of independent predictors, and the scale length of the nomogram variable is positively correlated with its impact on the prediction of PD‐MCI. Out of the three factors, the Rad‐score label contributed the most to predicting the outcome (the longest scale), followed by MoCA score. The high‐probability segment of the Rad‐score label corresponds to the high‐score area (score axis), and the low‐probability segment corresponds to the low‐score area. Patients with low MoCA scores had a higher probability of progressing to PD‐MCI than those with high scores. Patients with Symptom1 have an increased chance of progressing to PD‐MCI. The scores of all factors were added up to obtain the total score, which was perpendicular to (probability axis of progression to PD‐MCI) obtain the probability of individual final progression to PD‐MCI. The multimodal combinatorial model performed better than clinical features (MoCA score, Symptom1) in the training, test, and validation sets. The AUCs in the training, test, and validation sets were 0.842 (95% CI, 0.767–0.900), 0.829 (95% CI, 0.702–0.918), and 0.860 (95% CI,0.711–0.950), with sensitivities of 0.857, 0.733, and 0.889, respectively, and specificities of 0.745, 0.821, and 0.714, respectively (Figure 4B–D). The Hosmer–Lemeshow test showed that the multimodal combinatorial model did not overfit (*p* > 0.05), and the calibration curve showed that the predictive performance of the multimodal combinatorial model was consistent with the actual MCI progression state (Figure 4E–G). The Delong test showed that there was a significant difference (*p* < 0.05) in the diagnostic performance of the multimodal combinatorial model and the independent predictive factors MoCA and Symptom1 in the training, test, and validation sets, and there was no significant difference compared to the radiomics model (Table 3). Finally, The confusion matrix was used to describe the performance of the multimodal combinatorial model (Figure 5), the F1 score in the training, test, and validation sets were 0.627, 0.667, and 0.842, respectively.

**TABLE 2:** Screening of independent predictive factors.

Variable	Univariate logistic regression		Multivariate logistic regression	
	OR (95% CI)	*p*‐Value	OR (95% CI)	*p*‐Value
Age (year)	1.045 (0.991, 1.100)	0.099	NA	NA
Sex	0.948 (0.431, 2.087)	0.895	NA	NA
Educyrs	0.967 (0.848, 1.103)	0.618	NA	NA
Symptom1	2.947 (0.900, 9.648)	0.074	2.908 (0.892, 13.136)	0.108
Symptom2	1.478 (0.580, 3.767)	0.413	NA	NA
Symptom3	0.791 (0.285, 2.199)	0.653	NA	NA
Symptom4	1.249 (0.366, 4.258)	0.723	NA	NA
Symptom5	0.618 (0.212, 1.805)	0.379	NA	NA
Hy	1.275 (0.601, 2.704)	0.527	NA	NA
ESS	0.480 (0.151,1.529)	0.215	NA	NA
GDS	0.971 (0.253, 3.730)	0.966	NA	NA
RBD	1.688 (0.807, 3.531)	0.164	NA	NA
MoCA	0.697 (0.516, 0.944)	0.020*	0.627 (0.439, 0.876)	0.008**
Radiomics model	2.252 (1.518, 3.342)	<0.001***	2.166 (1.565, 2.998)	<0.001***

*Note*: NA, not available because the variable is not included in multiple variables. The *p* value indicates whether the variable is an independent predictor of PD‐MCI.

* *p* < 0.05.

** *p* < 0.01.

*** *p* < 0.001.

**TABLE 3:** Comparison of the diagnostic efficacy of the multimodal combinatorial model, radiomics model, MoCA scores, and Symptom1 in the training, test, and validation sets.

Characteristics	Training set				Test set				Validation set			
	AUC	Sensitivity	Specificity	*p*‐Value	AUC	Sensitivity	Specificity	*p*‐Value	AUC	Sensitivity	Specificity	*p*‐Value
Multimodal combinatorial model	0.842	0.857	0.745	NA	0.829	0.733	0.821	NA	0.860	0.889	0.714	NA
Radiomics model	0.819	0.743	0.766	0.290^a^	0.793	0.733	0.846	0.391^a^	0.828	0.889	0.667	0.657^a^
MoCA	0.666	0.571	0.713	*p* < 0.001^b^ ***	0.638	0.600	0.615	0.048^b^ *	0.642	0.722	0.524	0.002^b^ **
Symptom1	0.579	0.914	0.245	*p* < 0.001^c^ ***	0.582	0.933	0.231	*p* < 0.001^c^ ***	0.532	0.111	0.952	*p* < 0.001^c^ ***

*Note*: The markers a, b, and c represent the diagnostic performance comparison of the multimodal combinatorial model with the radiomics model, MoCA, and Symptom1, respectively.

Abbreviation: NA, not available.

* *p* < 0.05.

** *p* < 0.01.

*** *p* < 0.001.

### 3.4 Verification of the multimodal combinatorial model

**FIGURE 6:**
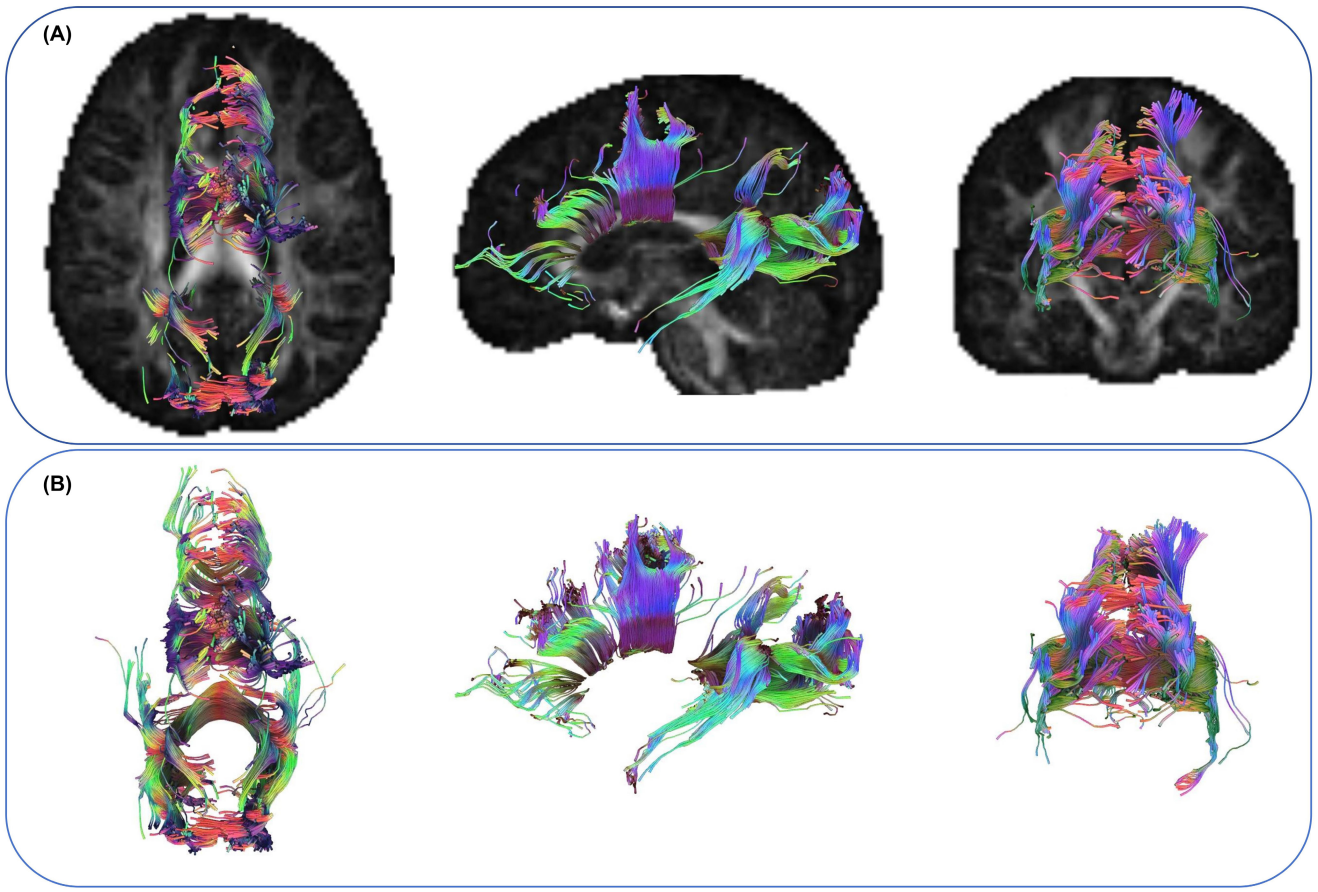
Representative patients with quantitative characteristics of DTI, tracking legend of corpus callosum fiber bundles. A and B show the specific location of the corpus callosum fiber bundle in the brain and the fully extracted fiber bundle morphology, respectively.

**FIGURE 7:**
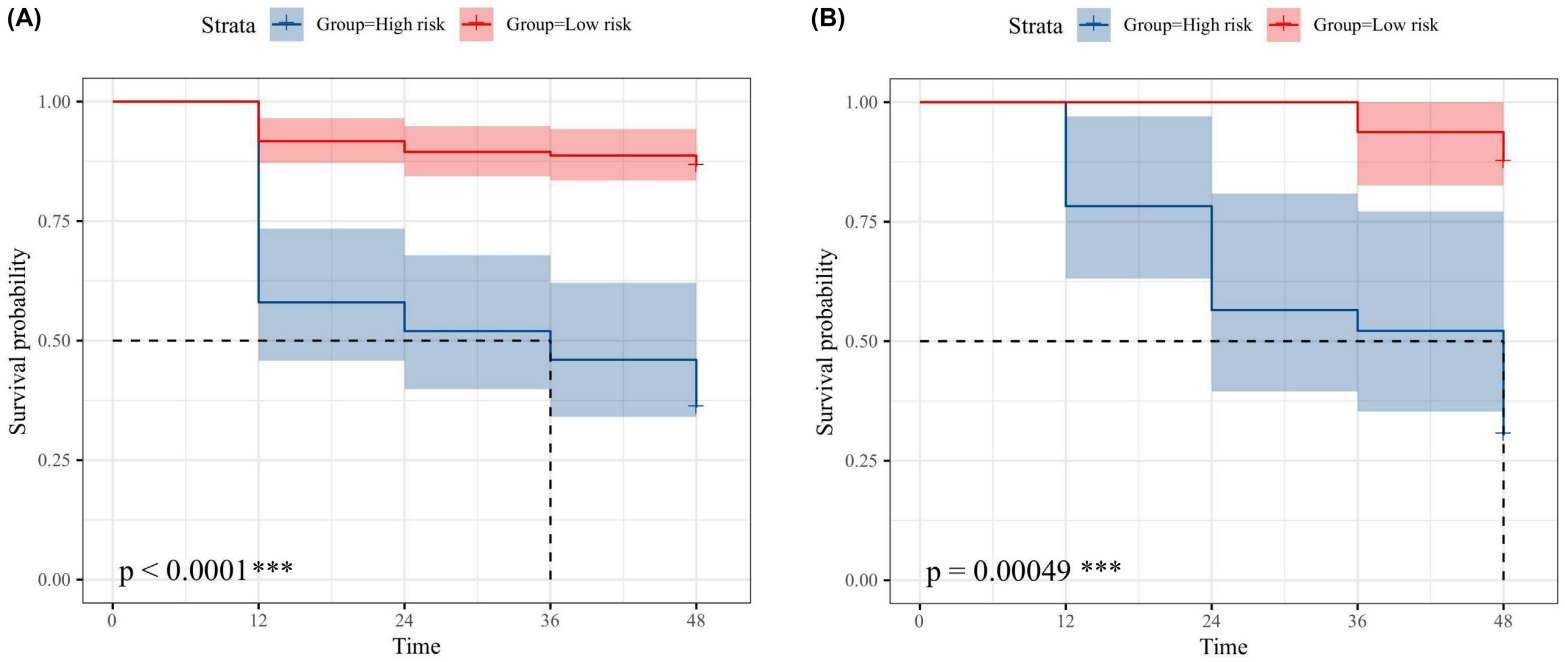
The survival curve analysis of low‐risk and high‐risk groups based on the multimodal combinatorial model classification in the PPMI (A) and NACC (B), respectively. ***p < 0.001.

Among 52 patients with DTI quantitative characteristics, the fractional anisotropy (FA), mean diffusivity (MD), and axial diffusion (AD) values of the corpus callosum fiber bundle showed significant differences in the low‐ and high‐risk groups, while the relative anisotropy (RD) values were not statistically significant. The same results were observed in the nonprogression and progression groups (Figure 6 and Table 4). Survival analysis was performed using the log‐rank test and showed statistically significant differences in MCI progression time between the low‐risk and high‐risk groups in the PPMI and NACC databases (Figure 7).

**TABLE 4:** DTI quantitative characteristics‐differences of the corpus callosum between groups.

Group (*n* = 52)	Four‐year follow‐up classification			Multimodal combinatorial model classification		
	Nonprogression group (*n* = 40)	Progression group (*n* = 12)	*p*‐Value	Low‐risk group (*n* = 37)	High‐risk group (*n* = 15)	*p*‐Value
FA, mean (SD)	0.289 ± 0.013	0.280 ± 0.012	0.021*	0.289 ± 0.013	0.286 ± 0.010	0.015*
MD, mean (SD)	1.037 ± 0.069	1.107 ± 0.063	0.001**	1.039 ± 0.073	1.074 ± 0.060	0.024*
AD, mean (SD)	1.313 ± 0.070	1.385 ± 0.063	0.001**	1.315 ± 0.074	1.353 ± 0.061	0.033*
RD, mean (SD)	0.916 ± 0.075	0.932 ± 0.079	0.397	0.917 ± 0.085	0.924 ± 0.050	0.579

*Note:* Data are presented as mean ± standard deviation.

* *p* < 0.05.

** *p* < 0.01.

## 4 DISCUSSION

This study used radiomic features from conventional magnetic resonance structural images to construct a radiomics model. We conducted a systematic and quantitative review of the prediction of clinical status evolution in PD‐NC individuals over 4 years. The results indicate that the predictive performance of the multimodal combinatorial model is significantly better than that of clinical features alone (MoCA score, Symptom1), indicating that a radiomics model based on the whole brain can be used to identify patients who progress from PD‐NC to PD‐MCI. In addition, the multimodal combinatorial model constructed by combining these feature types significantly improves the prediction performance. This may also provide a beneficial tool for the clinical screening of potentially high‐risk populations for PD‐MCI.

MRI, as a noninvasive and intuitive reflection of brain tissue structure, has become one of the essential measurement methods for PD research.^24^ T1‐based structural MRI methods, such as VBM analysis of several brain regions, including the hippocampus, have been widely used in studying PD evolution.^25^ However, these brain regions reflect only the local pathological mechanisms of the disease rather than the pathological changes that evolve from PD‐NC to PD‐MCI. In this study, we developed a new whole‐brain radiomics model to determine the high‐risk population of PD‐NC patients who may progress to PD‐MCI. Our research results indicate that the radiomics model has good diagnostic efficacy, which may reflect most pathological changes during disease progression. This study showed that 5 gray and 3 white matter features were involved in constructing the model. These 8 quantitative features may display pathological differences, which the original T1WI images cannot recognize. However, our analysis of the classification performance of our radiomic features model indirectly confirms the pathological changes in the brain between the high‐ and low‐risk groups. In addition, among the 8 features, there were 6 wavelet features, representing the texture's smoothness and heterogeneity. Previous studies by Mayerhoefer et al.^26^ revealed that higher wavelet eigenvalues are associated with rougher and more heterogeneous tissue structures. Therefore, the wavelet features in label construction in this study suggest that brain structural damage may lead to changes in the smoothness and heterogeneity of voxels in disease‐related regions, uncovering more profound pathological changes.

Our research results also show that using Symptom1, the MoCA score, and radiomics model alone can predict the risk of developing MCI, but these features also have certain limitations. Although Symptom1 is associated with the risk of MCI, not all individuals with static tremor will develop PD‐MCI,^27^ which may also be a possible reason for its poor specificity as a predictive factor. In evaluating cognitive function, MoCA is the most commonly used neuropsychological scale in clinical practice to quickly and easily assess cognitive function. However, it exhibits poor psychological measurement characteristics and low sensitivity and specificity,^28^ and as a subjective quantitative measure, it cannot directly reflect changes in neuropathology. In addition, although the diagnostic performance of radiomics model is close to that of a multimodal combinatorial model and can reflect some changes in neuropathology, due to the long course of PD. They cannot immediately reflect whether PD‐NC patients will progress to MCI. The comprehensive model constructed by combining the above three showed the highest diagnostic performance and achieved high sensitivity and specificity. This result is also similar to the research results of Sun et al.,^29^ who confirmed the significant application value of clinical features combined with MRI radiomic features for diagnosing PD‐MCI through a meta‐analysis of 32 cohort studies. Therefore, a multimodal combinatorial model using these features can more comprehensively and accurately reflect whether PD‐NC patients will progress to MCI, which may also provide new ideas and methods for the early identification of high‐risk PD patients who may progress to MCI.

Considering the lack of biological interpretability of radiomic features, DTI is a non‐invasive method for measuring microstructural changes in brain white matter,^30^ it can detect early white matter alterations in patients with PD.^31^ The results showed that the FA value in the corpus callosum area decreased in the high‐risk group compared to the low‐risk group, while the AD and MD values significantly increased in the low‐risk group. The same results were also observed in the nonprogression and progression groups. Bledsoe et al.^32^ showed that white matter abnormalities in the measurement of DTI in the corpus callosum region may lead to cognitive impairment in PD by interfering with information transmission projected between the cerebral hemispheres and the corpus callosum cortex. Gorges et al.^33^ showed slight changes in the structure of the corpus callosum in 206 patients with PD‐NC, while severe white matter damage was observed in the corpus callosum of PD‐MCI, manifested by a significant decrease in FA values and a significant increase in AD and MD values in the corpus callosum of PD‐MCI patients. This is consistent with the analysis results of our corpus callosum DTI, thus confirming the excellent diagnostic and classification performance of the multimodal combinatorial model.

This study also has certain limitations. First, it is a retrospective study with a small sample size, and larger samples and prospective studies are needed in the future. Second, due to the nonsimultaneous multicenter retrospective nature of the study, the MRI protocols of patients in the two centers were different (with different layer thicknesses), which may have led to more heterogeneity and bias in the study. However, we preprocessed the data to minimize the impact of MRI protocol differences. Finally, there is some uncertainty in this study's feature selection and modeling methods. The extraction of radiomic features has high dimensionality and complexity. Therefore, more research on selecting and combining these features and choosing appropriate modeling methods is still needed.

This study provides strong support for the prediction and diagnosis of MCI disease progression using radiomic features and helps to understand the mechanism of disease progression. At the same time, a multimodal combinatorial model based on radiomic features fills the gap in clinical screening of high‐risk MCI patients.

## AUTHOR CONTRIBUTIONS

YJJ and JXP conceptualized the study. YS, QWS, and TH collected the clinical data. JW, XYL, and JFC collected the MRI data. JXP, WW, and HS analyzed the data. YYX managed, validated, and visualized the data. YJJ wrote the manuscript. ZYS designed and supervised the whole research. All authors have read and agreed to the published version of the manuscript.

## FUNDING INFORMATION

The work was supported by the National Natural Science Foundation of China (Grant No. 82101983), the Department of Health of Zhejiang Province (Grant No. 2022500717), and the Zhejiang Provincial Natural Science Foundation of China (Grant No. LGF22H090021).

## CONFLICT OF INTEREST STATEMENT

The authors declare that the research was conducted in the absence of any commercial or financial relationships that could be construed as a potential conflict of interest.

## Supporting information


Data S1.

Data S2.

Figure S1.

Table S1.

## Data Availability

The data that support the findings of this study are available from the corresponding author upon reasonable request.
