# Supplementary material for: Prediction of cognitive decline in Parkinson's disease based on MRI radiomics and clinical features: A multicenter study
Source: CNS Neurosci Ther. 2024 Jun 24;30(6):e14789. doi: 10.1111/cns.14789 (PMC11196371; doi:10.1111/cns.14789)
Supplement: Supplementary file 1 — Data S1. [file CNS-30-e14789-s004.docx]

**Supplementary material**

DTI feature extraction.

DTI is an MRI technique based on fractional anisotropy (FA), mean diffusivity (MD), axial diffusion (AD), and relative anisotropy (RD) to evaluate changes in white matter microstructure, which can intuitively display changes in fiber connections between different brain regions[1]. Previous studies have shown that PD patients with normal cognition may have focal microstructural damage to the corpus callosum. PD-MCI patients exhibit severe white matter damage to the corpus callosum compared to PD-NC patients, characterized by a significant decrease in FA and an increase in MD and AD[2]. Based on the above research results, this study also extracted DTI parameters of the corpus callosum for analysis.

DTI image processing and analysis process: We use DSI Studio (https://dsi-studio.labsolver.org) to preprocess DTI images, including removing the skull from the image and performing manual checks to ensure that the removal of non-brain tissue is reasonable. Then, the image is processed through eddy current correction, head motion correction, and diffusion gradient direction adjustment. Finally, we mapped the white matter fiber bundle imaging pattern template to the standard ICBM152 space, segmented the image, selected the corpus callosum as the region of interest, and extracted and analyzed its DTI parameters, including FA, MD, AD, and RD.

**REFERENCES**

Beck D, de Lange AG, Maximov II, et al. White matter microstructure across the adult lifespan: A mixed longitudinal and cross-sectional study using advanced diffusion models and brain-age prediction. Neuroimage. 2021;224:117441.

1. Gorges M, Müller HP, Liepelt-Scarfone I, et al. Structural brain signature of cognitive decline in Parkinson's disease: DTI-based evidence from the LANDSCAPE study. Ther Adv Neurol Disord. 2019;12:1756286419843447. Published 2019 May 16.
