# Supplementary material for: Prediction of cognitive decline in Parkinson's disease based on MRI radiomics and clinical features: A multicenter study
Source: CNS Neurosci Ther. 2024 Jun 24;30(6):e14789. doi: 10.1111/cns.14789 (PMC11196371; doi:10.1111/cns.14789)
Supplement: Supplementary file 2 — Data S2. [file CNS-30-e14789-s003.docx]

**Supplementary material**

Detailed information on data dimensionality reduction.

1. Data standardization

Extracted radiomic features were standardized, which removed the unit limits of the data of each feature and converted it into a dimensionless pure value. This allowed the indexes of different units or orders to be compared and weighted. We used a z-score normalization to make the image intensities fit a standard normal distribution with $\mu\text{=0}$ and $\sigma\text{=1}$, where $\mu$ is the mean value of the images, and $\sigma$ is the standard deviation. The normalized values (also called z-scores) of the image intensities (*x*) were calculated as follows:

$$z=\frac{x-\mu}{\sigma}$$

1. Dimensionality reduction

A total of 3396 features were extracted from each patient, and detailed information on these features is shown in Table S1.

Firstly, we use the minimum redundancy maximum relevance (mRMR) algorithm to extract relevant features from the dataset. The minimum redundancy process ensures that the selected feature has minimal redundancy between other features. At the same time, the maximum relevance program selects the features with the most significant correlation with the progression of mild cognitive impairment. We select features with relevance coefficients greater than 0.8 and 0.1 as high correlation features and low redundancy features, respectively. Then, the mRMR method was used to obtain the optimal feature set with high relevance and low redundancy, retaining the remaining features: 20.

Finally, the least absolute shrinkage and selection operator (LASSO) method is used to reduce the dimensionality of the selected features. Lasso is a robust algorithm for regression analysis and high-dimensional prediction. The lasso algorithm reduces some coefficients through an absolute constraint and others to an exact zero. Therefore, LASSO is an excellent feature selection method because it uses subset selection and ridge regression, both retaining good features. In this study, 8 non-zero coefficients were selected for the lasso, and the details of the lasso are shown in Figure S2.

Using a logistic regression algorithm to evaluate the weight coefficients of various rad-scores, the formula is:

Rad-score=-0.723*Original_shape_Maximum2DDiameterColumn_gm

-0.171*Wavelet-LLL_glcm_ClusterProminence_wm

+1.051*Wavelet-LLH_gldm_LargeDependenceHighGrayLevelEmphasis_gm

-0.378*Original_firs-torder_Kurtosis_wm

-0.85*Wavelet-LHH_glcm_Imc1_gm

-0.748*Wavelet-HHL_first-order_Skewness_gm

+0.768*Wavelet-HHH_first-order_Mean_wm

-0.956*Wavelet-LLL_glszm_GrayLevelNonUniformity_gm

-0.723

A statistically significant differences in model scores between nonprogression and progression groups was observed for rad-scores in the training, test, and validation sets, as shown in Figure S3.

The final multimodal combinatorial model calculation formula is:

Multimodal combinatorial model score=8.16005014494653

+Symptom1*0.919544820673817

-MoCA*0.350327894268135

+Rad-score*0.659756140510234


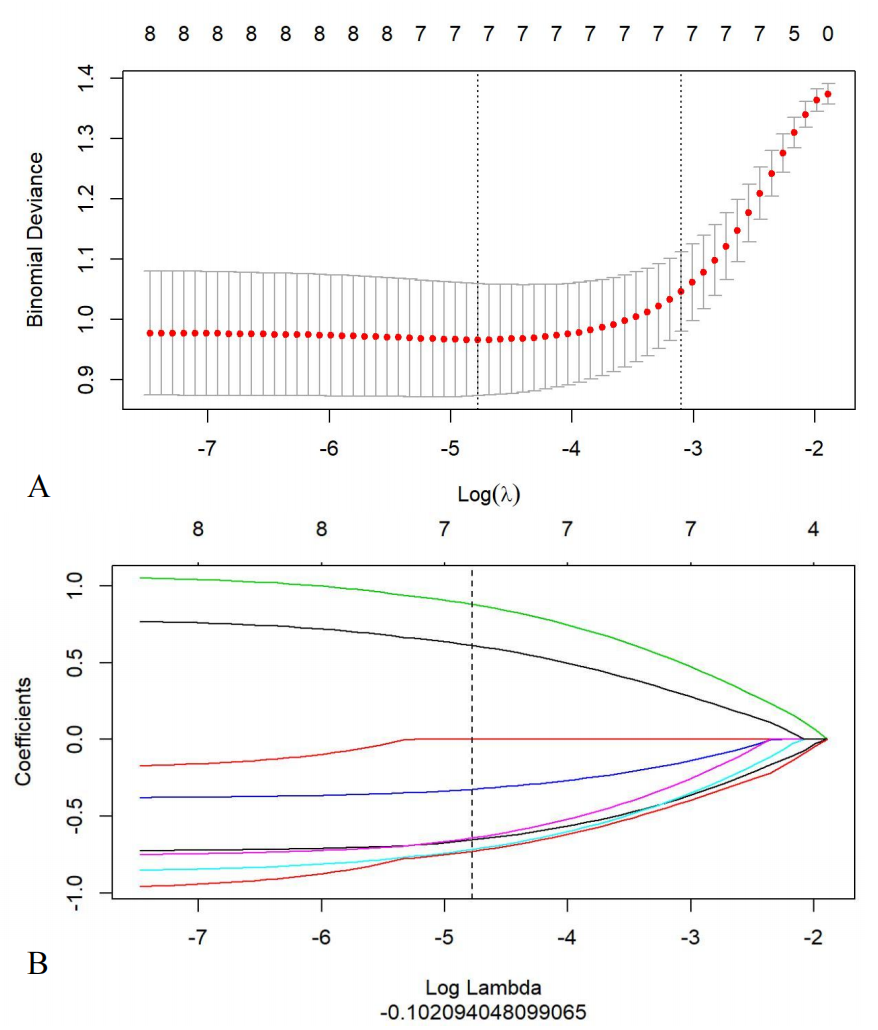


Figure S2 The feature selection during the lasso dimensionality reduction process in Figure A. Display the diagnostic bias of the model constructed after dimensionality reduction corresponding to different hyperparameters (input values). The vertical dashed line on the left represents the minimum deviation value corresponding to the optimal input value; The dashed line on the right represents the optimal input logarithmic function value. Figure B shows the changes in 8 features in different lasso systems with hyperparameter input values. The dashed line represents the 8 features with non-zero coefficients obtained.


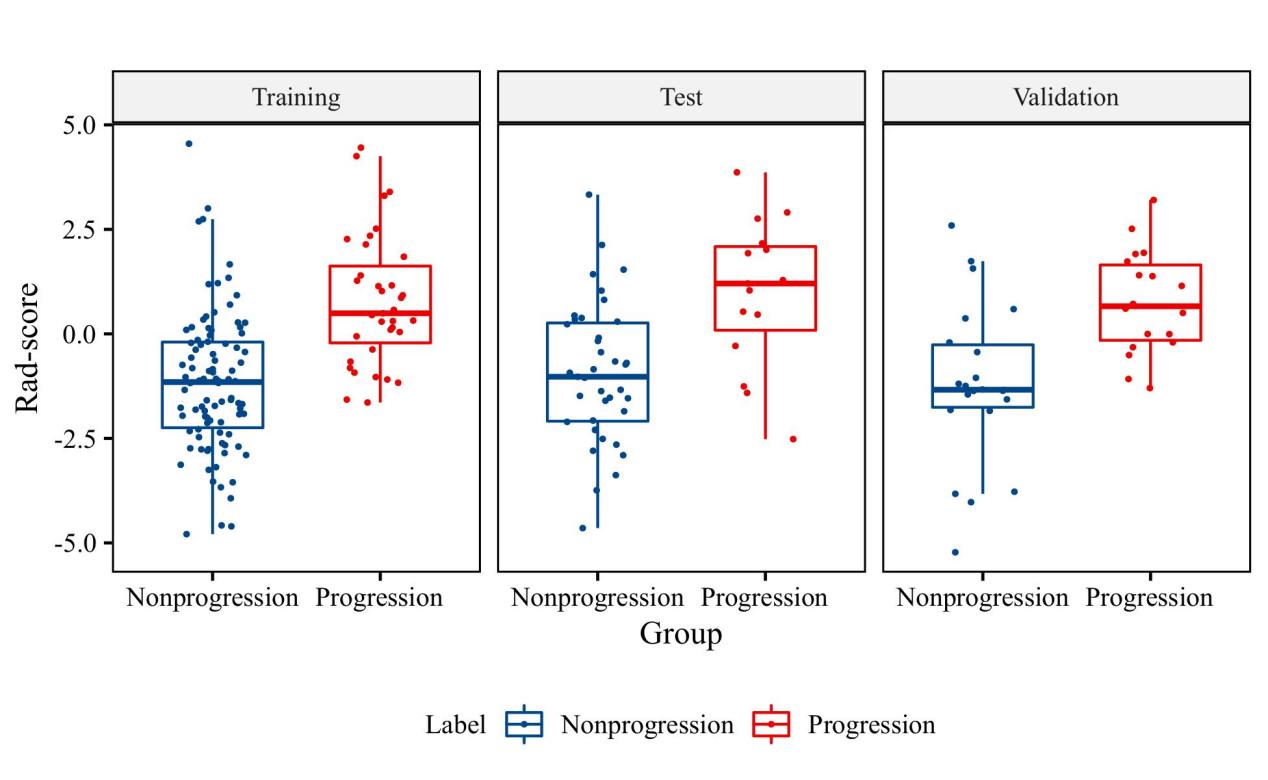


Figure S3 There are statistically significant differences in model scores between nonprogression and progression groups observed for rad-scores in the training, testing, and validation sets.
