# Supplementary figures and images for: Prediction of cognitive decline in Parkinson's disease based on MRI radiomics and clinical features: A multicenter study
Source: CNS Neurosci Ther. 2024 Jun 24;30(6):e14789. doi: 10.1111/cns.14789 (PMC11196371; doi:10.1111/cns.14789)

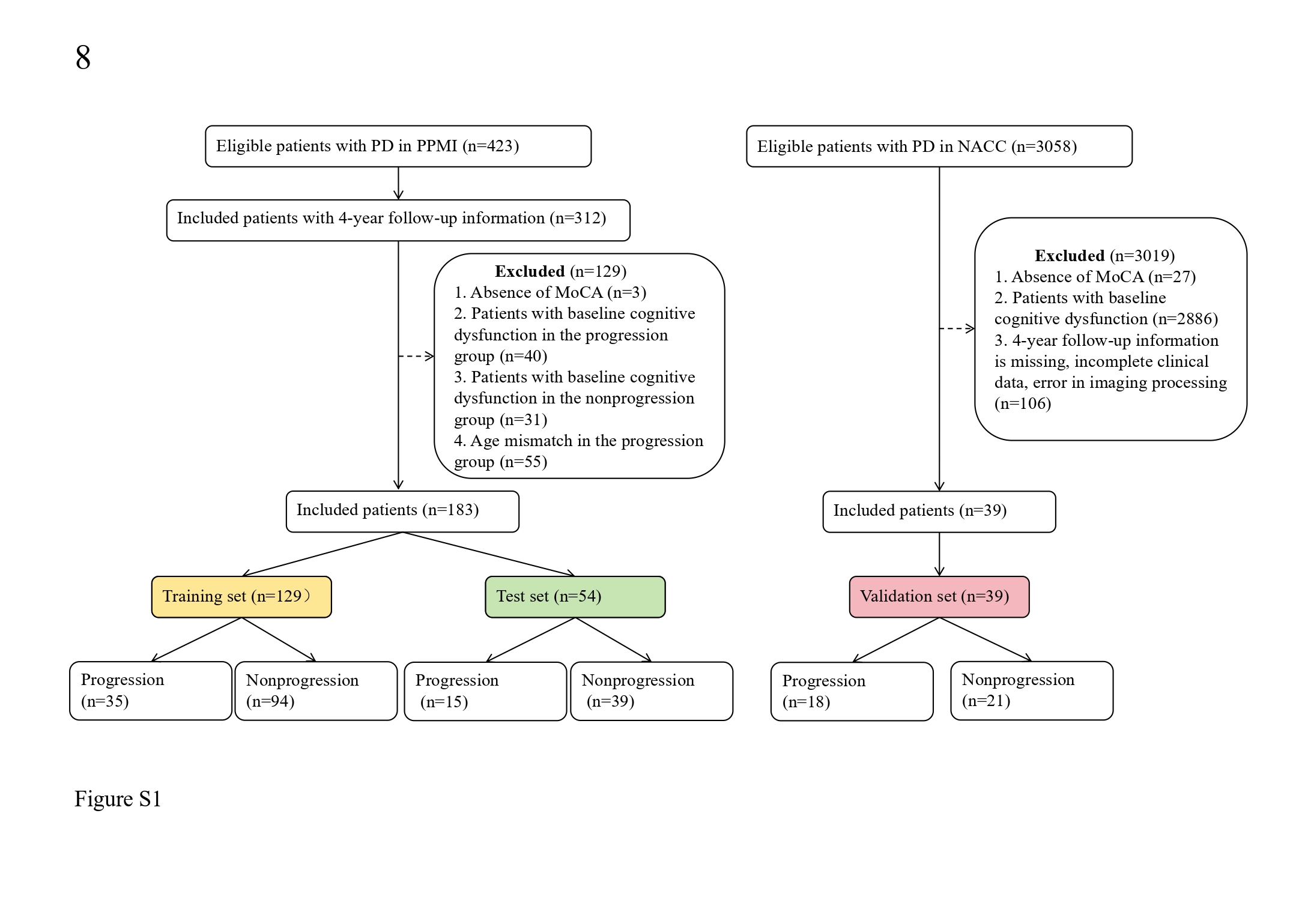

Supplement: Supplementary file 3 — Figure S1. [file CNS-30-e14789-s001.zip › Figure (1)_page-0008.jpg]

## Supplementary material

Figure S1 Flowchart of patient inclusion.

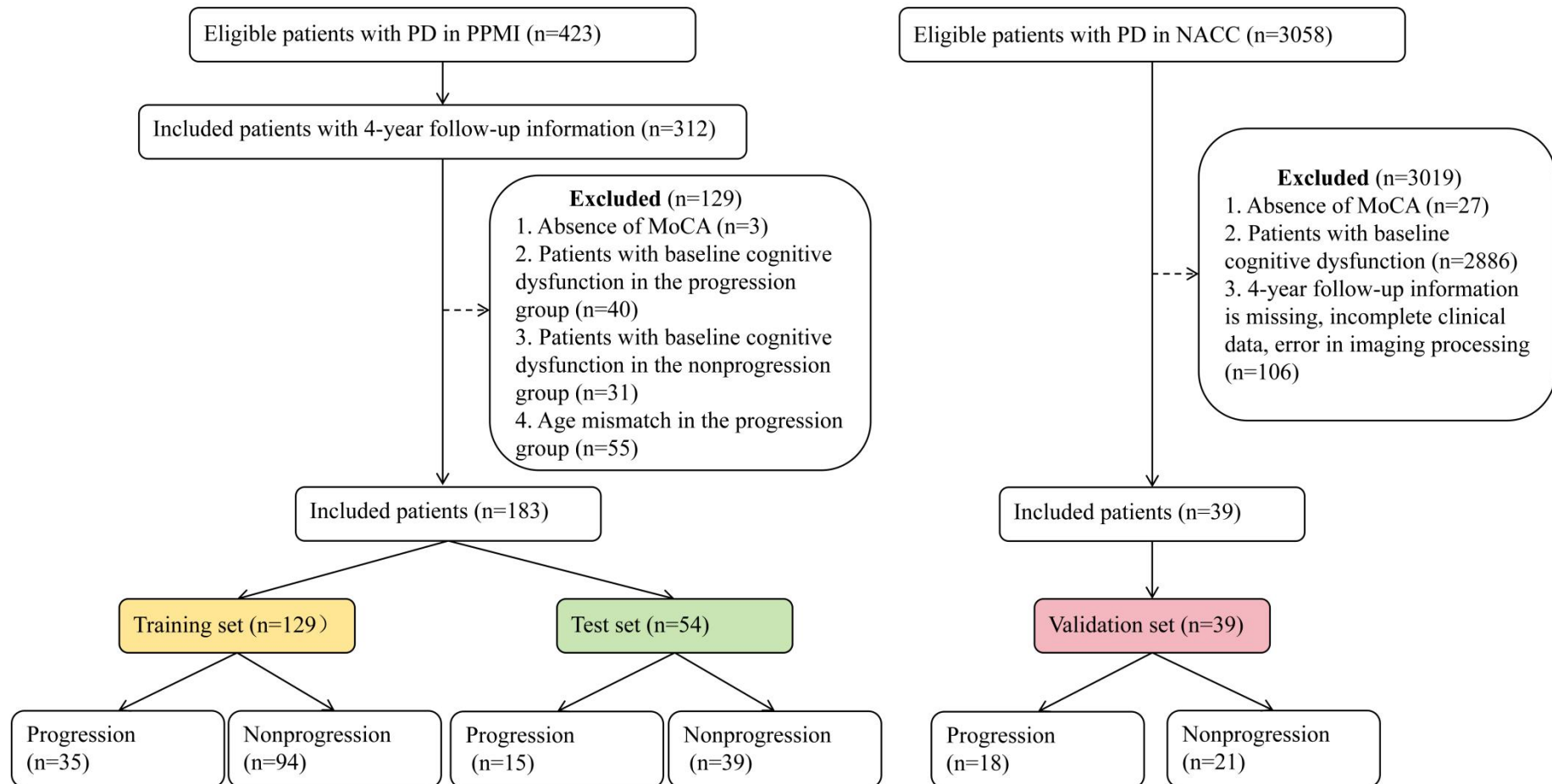

Supplement: Supplementary file 3 — Figure S1. [file CNS-30-e14789-s001.zip › Figure S1.pdf]

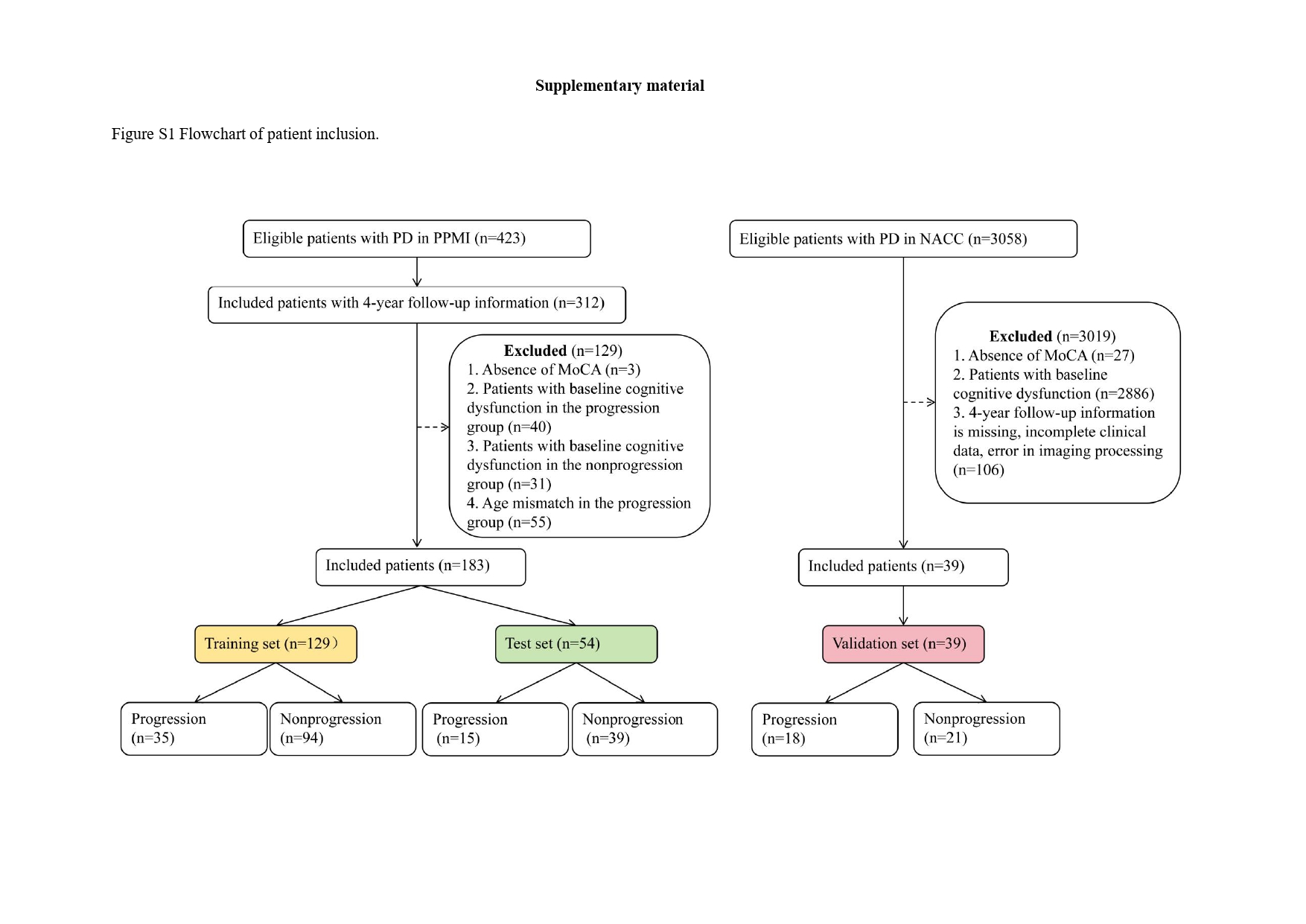

Supplement: Supplementary file 3 — Figure S1. [file CNS-30-e14789-s001.zip › Figure_S1_page-0001.jpg]
