# Supplementary material for: Prediction of cognitive decline in Parkinson's disease based on MRI radiomics and clinical features: A multicenter study
Source: CNS Neurosci Ther. 2024 Jun 24;30(6):e14789. doi: 10.1111/cns.14789 (PMC11196371; doi:10.1111/cns.14789)
Supplement: Supplementary file 4 — Table S1. [file CNS-30-e14789-s002.docx]

| **Class** | **Features** | **Class** | **Features** |
| --- | --- | --- | --- |
| **First-Order**  **Features**  (18 types) | 10Percentile | **Gray-Level Cooccurrence Matrix (GLCM) Features**  (22 types) | Autocorrelation |
|  | 90Percentile |  | ClusterProminence |
|  | Energy |  | ClusterShade |
|  | Entropy |  | ClusterTendency |
|  | InterquartileRange |  | Contrast |
|  | Kurtosis |  | Correlation |
|  | Maximum |  | DifferenceAverage |
|  | MeanAbsoluteDeviation |  | DifferenceEntropy |
|  | Mean |  | DifferenceVariance |
|  | Median |  | Inverse Difference (ID) |
|  | Minimum |  | Inverse Difference Moment (IDM) |
|  | Range |  | Inverse Difference Moment Normalized (IDMN) |
|  | RobustMeanAbsoluteDeviation |  | Inverse Difference Normalized (IDN) |
|  | RootMeanSquared |  | Informational Measure of Correlation (IMC) 1 |
|  | Skewness |  | Informational Measure of Correlation (IMC) 2 |
|  | TotalEnergy |  | InverseVariance |
|  | Uniformity |  | JointAverage |
|  | Variance |  | JointEnergy |
|  |  |  | JointEntropy |
|  |  |  | MaximumProbability |
|  |  |  | SumEntropy |
|  |  |  | SumSquares |
|  |  |  |  |

**Supplementary material**

**Table S1. Summary of extracted radiomic features.**

| **Class** | **Features** | **Class** | **Features** |
| --- | --- | --- | --- |
| **Gray-Level Run-Length Matrix (GLRLM) Features**  (16 types) | GrayLevelNonUniformity | **Gray-Level Size Zone Matrix (GLSZM) Features**  (16 types) | GrayLevelNonUniformity |
|  | GrayLevelNonUniformityNormalized |  | GrayLevelNonUniformityNormalized |
|  | GrayLevelVariance |  | GrayLevelVariance |
|  | HighGrayLevelRunEmphasis |  | HighGrayLevelZoneEmphasis |
|  | LongRunEmphasis |  | LargeAreaEmphasis |
|  | LongRunHighGrayLevelEmphasis |  | LargeAreaHighGrayLevelEmphasis |
|  | LongRunLowGrayLevelEmphasis |  | LargeAreaLowGrayLevelEmphasis |
|  | LowGrayLevelRunEmphasis |  | LowGrayLevelZoneEmphasis |
|  | RunEntropy |  | SizeZoneNonUniformity |
|  | RunLengthNonUniformity |  | SizeZoneNonUniformityNormalized |
|  | RunLengthNonUniformityNormalized |  | SmallAreaEmphasis |
|  | RunPercentage |  | SmallAreaHighGrayLevelEmphasis |
|  | RunVariance |  | SmallAreaLowGrayLevelEmphasis |
|  | ShortRunEmphasis |  | ZoneEntropy |
|  | ShortRunHighGrayLevelEmphasis |  | ZonePercentage |
|  | ShortRunLowGrayLevelEmphasis |  | ZoneVariance |
|  |  |  |  |

| **Class** | **Features** | **Class** | **Features** |
| --- | --- | --- | --- |
| **Gray-Level Dependence Matrix (GLDM) Features** (14 types) | DependenceEntropy | **Shape-Features**  **(**14 types**)** | Elongation |
|  | DependenceNonUniformity |  | Flatness |
|  | DependenceNonUniformityNormalized |  | LeastAxisLength |
|  | DependenceVariance |  | MajorAxisLength |
|  | GrayLevelNonUniformity |  | Maximum2DDiameterColumn |
|  | GrayLevelVariance |  | Maximum2DDiameterRow |
|  | HighGrayLevelEmphasis |  | Maximum2DDiameterSlice |
|  | LargeDependenceEmphasis |  | Maximum3DDiameter |
|  | LargeDependenceHighGrayLevelEmphasis |  | MeshVolume |
|  | LargeDependenceLowGrayLevelEmphasis |  | MinorAxisLength |
|  | LowGrayLevelEmphasis |  | Sphericity |
|  | SmallDependenceEmphasis |  | SurfaceArea |
|  | SmallDependenceHighGrayLevelEmphasis |  | SurfaceVolumeRatio |
|  | SmallDependenceLowGrayLevelEmphasis |  | VoxelVolume |
|  |  |  |  |
